# Antibodies against PM/Scl-75 and PM/Scl-100 are independent markers for different subsets of systemic sclerosis patients

**DOI:** 10.1186/ar2614

**Published:** 2009-02-16

**Authors:** Katharina Hanke, Claudia S Brückner, Cornelia Dähnrich, Dörte Huscher, Lars Komorowski, Wolfgang Meyer, Anthonia Janssen, Marina Backhaus, Mike Becker, Angela Kill, Karl Egerer, Gerd R Burmester, Falk Hiepe, Wolfgang Schlumberger, Gabriela Riemekasten

**Affiliations:** 1 Department of Rheumatology and Clinical Immunology, Charité Universitätsmedizin, Humboldt University Berlin, Charitéplatz 1, Berlin, 10117, Germany; 2 EUROIMMUN AG, Seekamp 31, Lübeck, 23560, Germany; 3 German Rheumatology Research Centre, Charitéplatz 1, Berlin, 10117, Germany

## Abstract

**Introduction:**

Anti-PM/Scl antibodies are present in sera from patients with polymyositis (PM), systemic sclerosis (SSc), and PM/SSc overlap syndromes. The prevalence of antibodies against the 75- and 100-kDa PM/Scl proteins and their clinical associations have not been studied in SSc patients in detail so far but could provide a valuable tool for risk assessment in these patients. Furthermore, it remains speculative whether commercially available test systems detecting only anti-PM/Scl-100 antibodies are sufficient in SSc patients.

**Methods:**

Two hundred eighty sera from SSc patients, patients with other connective tissue diseases (n = 209), and healthy blood donors (n = 50) were analyzed for the presence of anti-PM/Scl-75 and anti-PM/Scl-100 antibodies by means of line immunoblot assay. For the SSc patients, possible associations between both subsets of anti-PM/Scl antibodies with clinical and laboratory findings were studied.

**Results:**

The determination of anti-PM/Scl reactivity revealed a diagnostic sensitivity of 12.5% and a specificity of 96.9% for SSc. Among anti-PM/Scl-positive SSc patients, 10.4% and 7.1% were positive for anti-PM/Scl-75 and anti-PM/Scl-100 antibodies, respectively. The highest prevalences of reactivity to PM/Scl were detected in diffuse SSc (19.8%) and overlap syndromes (17.6%). Patients with diffuse SSc showed mainly an anti-PM/Scl-75 response, whereas most cases of overlap syndromes were characterized by reactivity to both PM/Scl antigens. The presence of anti-PM/Scl-75/100 antibodies was associated with muscular and lung involvements as well as with digital ulcers; pulmonary arterial hypertension was found less frequently. Anti-PM/Scl-75 antibodies were detected more frequently in younger and more active patients with joint contractures. Anti-PM/Scl-100 antibodies were associated with creatine kinase elevation; however, gastrointestinal involvements were observed less frequently.

**Conclusions:**

Anti-PM/Scl antibodies are common in distinct SSc subsets and are associated with several clinical symptoms. They are directed mainly to the PM/Scl-75 antigen. Consequently, the detection of anti-PM/Scl antibodies by tests based only on PM/Scl-100 as an antigen source may miss a relevant number of SSc patients positive for these antibodies.

## Introduction

Autoantibodies often characterize patients with distinct clinical features and often have prognostic relevance in different connective tissue diseases. Anti-PM/Scl antibodies, first described in patients with an overlap syndrome of polymyositis (PM) and scleroderma (systemic sclerosis [SSc]), seem to be rare antibodies, especially when SSc patients were studied [1]. In what is currently the largest study on the prevalence of anti-PM/Scl antibodies using the Pittsburgh Scleroderma Databank, only 2.5% of the SSc patients exhibited anti-PM/Scl antibodies [2]. The low number of anti-PM/Scl-positive patients did not allow conclusive analyses concerning associated clinical features, and the SSc patients were not classified according to their disease subsets. However, the descriptions of anti-PM/Scl-positive patients point to a higher prevalence of patients with muscular involvement, supporting other investigations using smaller populations or patients with myositis [1,3-6]. An association between the presence of anti-PM/Scl antibodies and Raynaud phenomenon (RP), arthritis, and interstitial lung disease was suggested as well [5].

Anti-PM/Scl antibodies are a heterogeneous group of autoantibodies directed to several proteins of the nucleolar PM/Scl macromolecular complex. The two main autoantigenic protein components were identified and termed PM/Scl-75 and PM/Scl-100 based on their apparent molecular weights [7,8]. According to former studies indicating PM/Scl-100 as the main target of the autoimmune response to PM/Scl, the majority of commercially available assays use recombinant PM/Scl-100 protein [3]. However, recent studies also suggest the diagnostic importance of anti-PM/Scl-75 antibodies, especially when the major isoform PM/Scl-75c is used as an antigen source [9,10]. The percentage of patients presenting anti-PM/Scl-75c antibodies is supposed to exceed that for anti-PM/Scl-100 antibodies [9]. However, analyses of larger SSc cohorts to identify the prevalence and specificity of these antibodies are missing. Furthermore, it remains elusive whether the different antibodies reflect different SSc subsets and clinical features present in these patients.

Based on the growing knowledge about the anti-PM/Scl antibody targets, very sensitive methods such as an enzyme-linked immunosorbent assay (ELISA), which is based on a PM/Scl-100-derived peptide called PM1-alpha, have been developed [11]. In recent years, line immunoblot assay (LIA) has become a popular technique for the simultaneous detection of several autoantibodies. As recently shown and exemplified for the determination of anti-topoisomerase I (anti-topo I) antibodies, LIA provides a valuable tool as an alternative to ELISA [12].

In the present study, a large monocentric cohort of consecutive SSc patients was analyzed by LIA, allowing the simultaneous monospecific detection of both anti-PM/Scl-75 and anti-PM/Scl-100 antibodies. Clinical data were assessed simultaneously by a standardized procedure with only a limited number of investigators. For patient assessment, we applied criteria and strategies developed by the German Network of Systemic Scleroderma (DNSS) and the European Scleroderma Trials and Research (EUSTAR) network [13-15]. By this approach, we identified several clinical features associated with the presence of either anti-PM/Scl antibody.

## Materials and methods

### Classification of patients

Sera from 280 consecutive SSc patients were analyzed for the presence of anti-PM/Scl antibodies. Patients were divided into different subsets according to the criteria of the EUSTAR and DNSS network [13,14]. Briefly, diffuse SSc (dSSc) and limited SSc (lSSc) were defined according to LeRoy and colleagues [16] and the DNSS and EUSTAR criteria based on the maximal distribution of skin involvement during the disease course. Overlap syndromes, including mixed connective tissue disease, were defined as a disease occurring with clinical aspects of SSc or main symptoms of SSc in parallel to those of other connective tissue diseases [17]. SSc sine scleroderma (SScSS) was defined as described by Rodnan and Fennel [18]. Undifferentiated connective tissue disease (UCTD) with scleroderma features was defined as positive RP and at least one further feature of SSc (for example, typical nail fold capillary alterations, puffy fingers, or pulmonary hypertension) and/or detectable scleroderma-associated autoantibodies without fulfilment of American College of Rheumatology criteria [19]. By using (or applying) the criteria of LeRoy and colleagues [16], these patients can also be classified as having limited disease. According to these criteria, our study included 113 patients with lSSc, 96 patients with dSSc, 51 patients with an overlap syndrome, 16 patients with UCTD, and 4 patients with SScSS. The clinical and epidemiological data of this cohort are presented in Table 1. As demonstrated before, our cohort is representative of European SSc cohorts showing similar clinical features [12]. In addition, 259 control sera from patients with Sjögren syndrome (SS) (n = 49), systemic lupus erythematosus (SLE) (n = 72), and rheumatoid arthritis (RA) (n = 88) and from healthy blood donors (n = 50) were analyzed. All control patients were diagnosed according to internationally recognized criteria [20-22].

**Table 1:** Clinical and demographic characteristics of the Charité cohort

	dSSc	lSSc	SScSS	Overlap	UCTD	All
Number	96	113	4	51	16	280
Age in years^a^	54.5 (14.7)	63.0 (11.2)	52.5 (12.9)	51 (13.2)	55.5 (10.6)	58.0 (13.3)
Duration of RP in years^a^	5 (10.2)	9.5 (12.6)	12 (21.3)	7 (8.6)	8 (9.6)	7.5 (11.3)
Duration of disease in years^a^	5 (7.7)	8 (7.0)	3.5 (6.8)	7 (7.6)	5 (10.9)	7 (7.6)
Females/males	79/17	105/8	2/2	41/10	16/0	243/37
mRSS^a^	11.0 (9.61)	4.0 (4.3)	1.0 (1.1)	3.0 (6.9)	0.0 (1.1)	5.0 (8.0)
Digital ulcers^b^	52 (54.2)	38 (33.6)	2 (50)	18 (35.2)	2 (12.5)	112 (40.0)
Lung fibrosis^b^	57 (59.4)	16 (14.2)	3 (75)	20 (39.2)	2 (12.5)	98 (35.0)
DLCO-SB^a^, %	65.3 (21.9)	78.9 (17.5)	51.1(12.8)	71.2 (21.7)	80.0 (17.1)	72.4 (20.5)
Mean FVC^a^, %	82.1 (18.9)	97.4 (15.2)	95.5 (30.7)	86.5 (20.5)	95.2 (18.0)	91.6 (19.0)
Contractures^b^	78 (81.3)	62 (54.9)	2 (50)	27 (52.9)	3 (18.8)	172 (61.4)
PAH^b^	22 (22.9)	22 (19.5)	2 (50)	13(25.7)	1 (6.3)	60 (21.4)
Renal involvement^b^	17 (17.7)	22 (19.5)	1 (25)	14 (27.5)	2 (12.5)	56 (20.0)
Renal crisis^b^	10 (10.4)	3 (2.7) n = 112	1 (25)	1 (2.0)	1 (6.3)	16 (5.7) n = 279
Cardiac involvement^b^	47 (49.0)	35 (31.0)	3 (75)	25 (49)	4 (25)	117 (40.7)
Skin involvement^b^	94 (98.9)	105 (92.9)	3 (75)	39 (76.5)	7 (43.8)	248 (88.9)
RP^b^	95 (99)	110 (97.3)	4 (100)	50 (98)	13 (81.3)	272 (97.1)

^a^Values displayed are medians (standard deviations). ^b^Values displayed are numbers of patients (percentages). DLCO-SB, predicted diffusion capacity of a single breath; dSSc, diffuse systemic sclerosis; FVC, forced vital capacity; lSSc, limited systemic sclerosis; mRSS, modified Rodnan skin score; PAH, pulmonary arterial hypertension; RP, Raynaud phenomenon; SScSS, systemic sclerosis sine scleroderma; UCTD, undifferentiated connective tissue disease.

### Assessment of the systemic sclerosis patients

Between January 2004 and May 2007, sera of 280 SSc patients were collected during the clinical assessment of the patients, stored at -20°C, and analyzed for the presence of SSc-associated antibodies. Most patients were assessed by one investigator (GR), who instructed the only other investigator (CSB). Both investigators participated in several training programs of EUSTAR and DNSS for the assessment of patients. In general, 26 clinical and laboratory findings were assessed and analyzed as described [12-14]. Briefly, for the evaluation of fibrotic skin changes and for the classification of the SSc subsets, the modified Rodnan skin score (mRSS) was used [23]. Pulmonary arterial hypertension (PAH) was defined when assessed by a right heart catheter with mean pulmonary arterial pressures of 25 mm Hg at rest and 30 mm Hg while exercising or by the presence of a pulmonary arterial systolic pressure of greater than or equal to 40 mm Hg and signs of right heart failure as detected by echocardiography. Pulmonary fibrosis was defined by evidence of fibrosis such as bibasilar fibrosis on chest radiogram and/or by high-resolution computed tomography scans. Lung function was assessed by the predicted forced vital capacity (FVC) and the predicted diffusion capacity of a single breath (DLCO-SB) method. Digital ulcers were defined as a loss of both epidermis and dermis in an area of at least 2 mm in diameter at the distal phalanx of fingers. Elevation of serum creatine kinase (CK) levels was considered when they increased above normal values. Disease activity was assessed by using the criteria of the European Scleroderma Study Group [24]. The study was approved by the local ethics committee (EA1/013/705). Written informed consent was obtained from each patient.

### Antibody detection by line immunoblot assay

For the detection of the different anti-PM/Scl antibodies, a profile LIA was developed and provided by EUROIMMUN AG (Lübeck, Germany). Briefly, recombinant PM/Scl-100 antigen was expressed by *Escherichia coli *or by baculovirus, spanning the major alpha helical epitope region between 231 and 245 (as described elsewhere [25]). PM/Scl-75c was expressed by baculovirus [10]. After affinity purification, the antigens were separately coated as lines onto nitrocellulose membrane chips that were fixed onto a plastic strip, creating a line immunoassay format based on the main target antigens of anti-PM/Scl antibodies. The LIA was additionally coated with antigens allowing anti-topo I, anti-U1-RNP, and anti-centromere protein-A/B (anti-CENP-A/B) antibody detection. To ensure diagnostic reliability and precision, the LIA was subjected to an extensive validation process. Sera from 280 SSc patients as well 259 controls were incubated according to the instructions of the manufacturer (EUROIMMUN AG) (30-minute serum incubation, washing step 1, 30-minute incubation with anti-human IgG/alkaline phosphatase, washing step 2, and 10-minute substrate incubation with NBT/BCIP [nitroblue tetrazolium/5-bromo-4-chloro-3-indolyl-phosphate]). Blot strips were digitalized using a flatbed scanner, and band intensities were evaluated by a computer program (EUROLineScan, EUROIMMUN AG). Signal strengths of greater than 6 units (U) were considered positive, as recommended by the manufacturer. All serological analyses were performed blindly by personnel unaware of the diagnosis or the clinical characteristics of the patients.

### Statistical analysis

The dataset was analyzed by means of the SPSS version 15.0 statistical package (SPSS Inc., Chicago, IL, USA) and the calculation software Excel version 12 (2007) (Microsoft Corporation, Redmond, WA, USA). For the analysis of qualitative values, chi-square and Fisher's exact tests were used. Quantitative values were compared by using the Mann-Whitney *U *test. *P *values of less than 0.05 were considered statistically significant.

## Results

### Prevalences of anti-PM/Scl-75 and anti-PM/Scl-100 in systemic sclerosis patients depend on disease subset and antigen expression system

**Figure 1:**
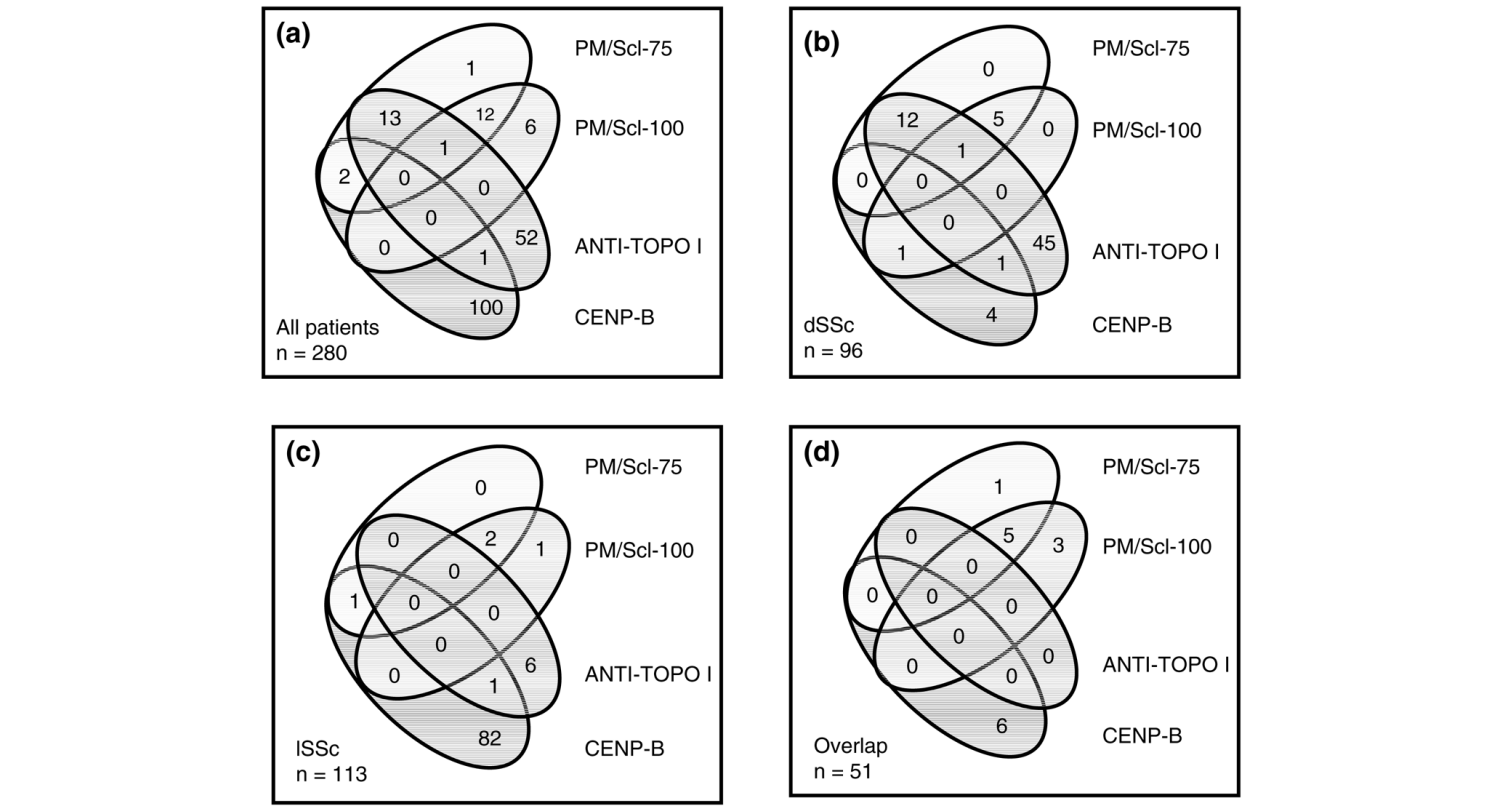
The presence of different anti-PM/Scl antibodies depends on the underlying disease subset in systemic sclerosis (SSc). Co-occurrence of antibodies that recognize different recombinant antigens as detected by line immunoblot assay in all anti-PM/Scl-positive patients **(a)**, in patients with diffuse SSc (dSSc) **(b)**, in patients with limited SSc (lSSc) **(c)**, and in patients with overlap syndromes **(d)**. anti-topo I, anti-topoisomerase I; CENP-B, centromere protein-B; PM, polymyositis.

Anti-PM/Scl-75 antibodies were present in 29 SSc patients (10.4%). Antibody reactivity against the PM/Scl-100 antigen expressed by *E. coli *was detected in 20 SSc patients (7.1%). All together, 35 out of 280 patients tested positive for anti-PM/Scl antibodies (12.5%) (Figure 1a). When the PM/Scl-100 antigen expressed by baculovirus was used, only 11 patients (3.9%) showed reactivity. The highest prevalences of anti-PM/Scl antibodies were found in dSSc patients (19.8%) and in patients suffering from overlap syndrome (17.6%) (Figure 1b,d). In contrast, anti-PM/Scl antibodies were rarely found in patients with lSSc (3.5%) (Figure 1c). In our control group with autoimmune diseases other than SSc (n = 259), there were eight anti-PM/Scl-positive sera with non-overlapping reactivities: On the one hand, reactivity to PM/Scl-100 (*E. coli*) was found in 1 out of 49 patients with SS (2%) and in 3 out of 72 patients with SLE (4.2%). On the other hand, anti-PM/Scl-75 antibodies were present in 1 patient with SLE (1.4%) and in 3 out of 88 patients (3.4%) with RA. None of the healthy blood donors exhibited either of these antibodies. Therefore, anti-PM/Scl antibody detection revealed an overall specificity for SSc of 96.9%. The specificities of anti-PM/Scl-75 and anti-PM/Scl-100 antibodies amounted to 98.5% each. The detection of antibodies directed to the PM/Scl-100 antigen expressed by baculovirus showed 100% specificity for SSc patients.

### Concordance of anti-PM/Scl-75, anti-PM/Scl-100, anti-topo I, and anti-CENP-B antibodies

For further analyses, we included only the results from LIA using PM/Scl-100 antigen expressed by *E. coli *and PM/Scl-75 autoantigen expressed by baculovirus. When the 35 anti-PM/Scl antibody-positive sera were analyzed for their subspecificities (anti-PM/Scl-75 and anti-PM/Scl-100) and for further autoantibodies, only one patient was exclusively positive for anti-PM/Scl-75 and six patients showed single reactivity to PM/Scl-100 (Figure 1a). Thirteen sera showed reactivity to both the PM/Scl-75 and the PM/Scl-100 antigen, and one of these sera was additionally reactive to topo I. A combination of anti-PM/Scl-75 and anti-topo I antibodies was found in another 13 serum samples. Two patients were positive for anti-CENP-B and anti-PM/Scl-75 antibodies. Two patients with anti-PM/Scl-75 antibodies also exhibited anti-U1-RNP antibodies (data not shown). When the subset of dSSc patients was studied (Figure 1b), 18 out of 19 anti-PM/Scl-positive sera revealed reactivity to PM/Scl-75 (94.7%). Among these 18 sera, 12 specimens were positive for both anti-PM/Scl-75 and anti-topo I antibodies, whereas the remaining 6 were reactive to both PM/Scl-75 and PM/Scl-100 (including only 1 anti-topo I-positive specimen). Only 1 out of 19 anti-PM/Scl-positive sera from dSSc patients showed reactivity to only PM/Scl-100, and this serum was also positive for antibodies directed to CENP-B. In patients with lSSc, 1 out of 4 anti-PM/Scl-positive patients showed positivity for PM/Scl-100 only, 2 patients were reactive to both PM/Scl-75 and PM/Scl-100, and 1 patient with sole anti-PM/Scl-75 antibodies also exhibited reactivity to CENP-B (Figure 1c). In overlap syndromes, 8 out of 9 sera with reactivity to PM/Scl contained anti-PM/Scl-100 antibodies, including 5 sera showing overlapping PM/Scl-75/100 reactivity. Only one patient was exclusively positive for anti-PM/Scl-75 (Figure 1d).

### Patients with overlap syndromes showed the highest signal strengths for the detection of anti-PM/Scl-75 antibodies

**Figure 2:**
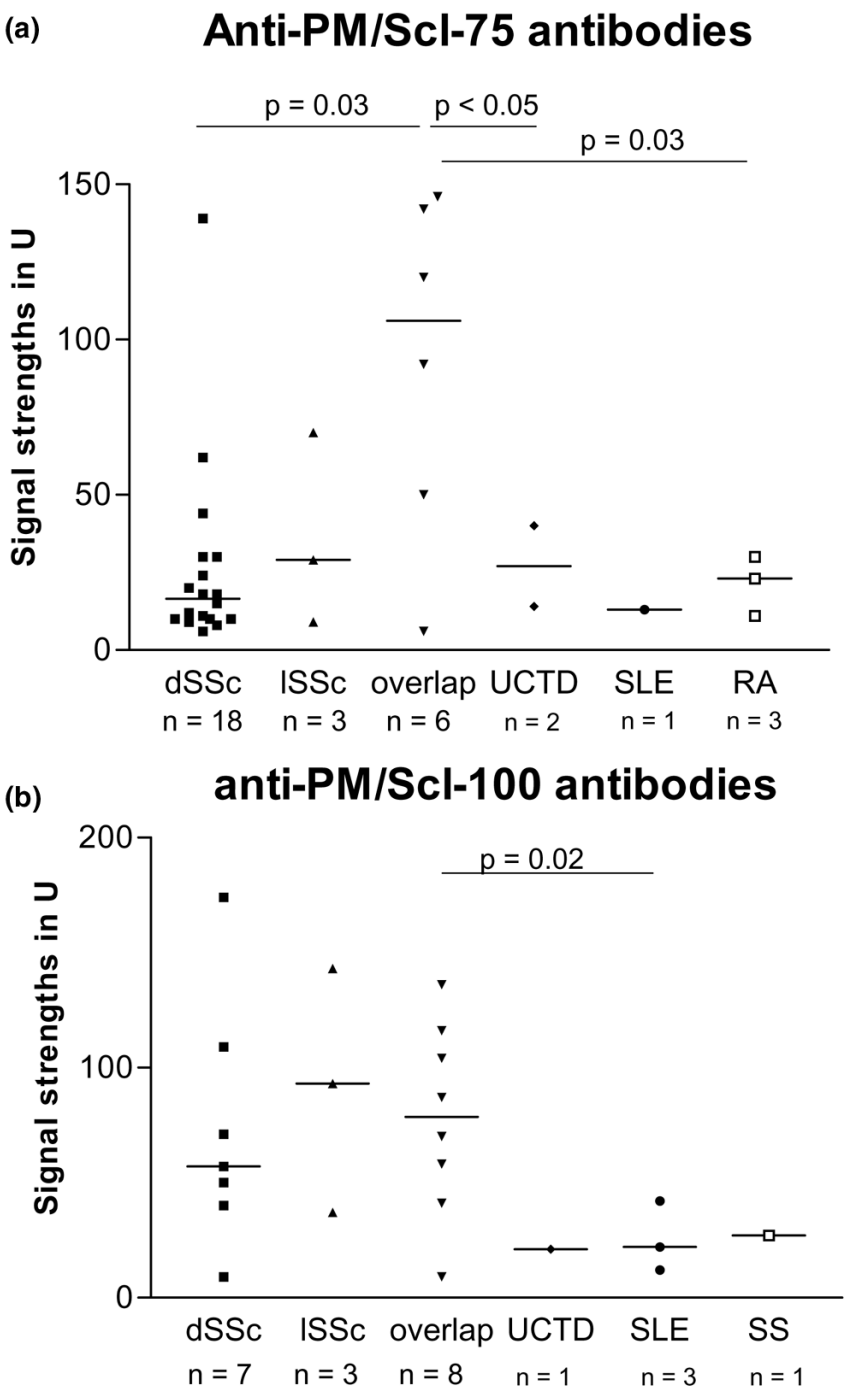
Signal strengths of anti-PM/Scl-75 antibodies **(a)**, but not of anti-PM/Scl-100 antibodies **(b)**, depend on the underlying disease. Sera from patients with diffuse systemic sclerosis (dSSc), limited systemic sclerosis (lSSc), overlap syndromes, and undifferentiated systemic sclerosis patients were analyzed by line immunoblot assay. PM, polymyositis; RA, rheumatoid arthritis; SLE, systemic lupus erythematosus; SS, Sjögren syndrome; U, units; UCTD, undifferentiated connective tissue disease.

The signal strengths of anti-PM/Scl-75 antibody-positive patients appeared to be related to the underlying disease and were highest in patients with overlap syndromes (Figure 2). Here, the median signal strength was 92.7 U and the signal strengths were significantly higher compared with those found in patients with dSSc, UCTD, or RA. Signal strengths of greater than 70 U were found in overlap syndromes nearly exclusively. Only one patient with dSSc who suffered from muscle pain and muscle atrophy without a detectable elevation of CK exhibited also a high signal strength of anti-PM/Scl-75 antibodies. In patients with dSSc and lSSc, the mean signal strengths were 27.6 and 37 U, respectively. Undifferentiated SSc revealed signal strengths similar to those observed in lSSc (Figure 2a). In an analysis of the signal strengths for the detection of anti-PM/Scl-100 antibodies, overlap syndromes did not show the highest values and the different disease subsets exhibited similar signal strengths (Figure 2b).

### Anti-PM/Scl antibodies were associated with muscle and lung involvement and were rarely found in patients with pulmonary arterial hypertension

In general, patients positive for anti-PM/Scl-75 and/or anti-PM/Scl-100 antibodies (n = 35) suffered significantly more frequently from digital ulcers and lung fibrosis compared with the anti-PM/Scl antibody-negative group (*P *= 0.005 and 0.004, respectively). Anti-PM/Scl-positive patients were also characterized by a higher prevalence of CK elevation (*P *= 0.002) and a significantly lower frequency of PAH (*P *= 0.049). There were no associations between the presence of anti-PM/Scl antibodies and skin, heart, and kidney involvement as well as neuropathies or sicca syndrome (data not shown). Patients with anti-PM/Scl antibodies did not receive more or less immunosuppressants than patients without these antibodies. Furthermore, no differences between antibody-positive and -negative patients were found by analyzing the gender ratio and the presence of a family history. No significant associations between the presence of anti-PM/Scl antibodies and mortality could be detected. Four out of 35 SSc patients died (11.4%). Correlations between the signal strengths of anti-PM/Scl antibodies and the degree of skin or lung fibrosis, mRSS, DLCO-SB, or FVC were not found (data not shown).

### The presence of anti-PM/Scl-75 antibodies identifies a distinct subtype of patients

Patients positive for anti-PM/Scl-75 antibodies revealed a significantly higher frequency (65.5%) of present or past digital ulcers compared with anti-PM/Scl-75-negative patients (37.1%; *P *= 0.005) (Table 2). Furthermore, the mean mRSS was considerably higher in the anti-PM/Scl-75-positive patients (9, standard deviation [SD] 11.3) than in the anti-PM/Scl-75-negative individuals (5, SD 7.4; *P *= 0.017). Patients with anti-PM/Scl-75 antibodies presented a higher prevalence of lung involvement also. Lung fibrosis could be found in 55.2% of the anti-PM/Scl-75-positive patients but in only 32.7% of the patients without this antibody (*P *= 0.023). In line with this, 62.1% of the anti-PM/Scl-75-positive patients suffered from restrictive lung disease, in contrast to 32.3% of the anti-PM/Scl-75-negative patients (*P *= 0.001). Furthermore, significant differences in the mean FVC values were detected (*P *< 0.005) by lung function tests. PAH occurred rarely in patients with PM/Scl-75 reactivity compared with the antibody-negative group (*P *= 0.054). Concerning the involvement of the musculoskeletal system, anti-PM/Scl-75-positive patients demonstrated a higher frequency of joint contractures and muscular atrophy than anti-PM/Scl-75-negative patients (*P *= 0.044 and 0.032, respectively). The prevalence of CK elevation was also higher in the anti-PM/Scl-75-positive group (*P *= 0.002). The EUSTAR activity score of anti-PM/Scl-75-positive patients was significantly higher compared with anti-PM/Scl-75-negative patients (*P *= 0.037). The onset of disease in the anti-PM/Scl-75-positive patients was at a mean age of 44.2 years (SD 17.6 years), which was 6 years earlier than in the anti-PM/Scl-75-negative patients (mean age 50.4 years, SD 13.8 years; *P *= 0.057).

**Table 2:** Comparison between anti-PM/Scl-75 antibody-positive versus-negative patients

Disease manifestation	anti-PM/Scl-75-positive (n = 29)	anti-PM/Scl-75-negative (n = 251)	*P *value	Sensitivity, percentage^a^	Specificity, percentage^b^
mRSS^c^	9.0 (11.3)	7.2 (7.4)	0.017	NA	NA
Digital ulcers^d^	19 (65.5)	93 (37.1)	0.005	17	94
Lung fibrosis^d^	16 (55.2)	82 (32.7)	0.023	16.3	92.9
Restrictive lung disease^d^	18 (62.1)	81 (32.3)	0.001	18.2	94.8
DLCO-SB^c^	65.7 (18.1)	73.7 (20.7)	0.091	NA	NA
Mean FVC^c^	80.0 (14.1)	93.6 (19.2)	<0.005	NA	NA
PAH^d^	2 (6.9)	58 (23.1)	0.054	3.3	87.7
Contractures^d^	23 (79.3)	149 (59.4)	0.044	13.4	94.4
CK elevation^d^	10 (34.5)	27 (10.8)	0.002	27	92.2
Muscle atrophy^d^	19 (65.5)	111 (44.2)	0.032	14.6	93.3
Colon involvement^d^	7(24.1)	112 (44.6)	0.046	5.9	86.3
Disease activity^c^	2.0 (1.2)	1.5 (1.3)	0.037	NA	NA

Clinical differences between antibody-positive and -negative patients were shown only when *P *values of below 0.1 were detected. ^a^Probability that patients with the respective disease manifestation are anti-PM/Scl-75-positive; for example, sensitivity of anti-PM/Scl-75 (digital ulcers) = 19/(19 + 93) = 0.17. ^b^Probability that patients without the respective disease manifestation are anti-PM/Scl-75-negative; for example, specificity of anti-PM/Scl-75 (digital ulcers) = (251 - 93)/(29 - 19 + 251 - 93) = 0.94. ^c^Values displayed are medians (standard deviations). ^d^Values displayed are numbers of patients (percentages). CK, creatine kinase; DLCO-SB, predicted diffusion capacity of a single breath; FVC, forced vital capacity; mRSS, modified Rodnan skin score; NA, not applicable; PAH, pulmonary arterial hypertension.

### Anti-PM/Scl-100 antibodies were associated with fewer gastrointestinal symptoms

Patients with antibodies to PM/Scl-100 (*E. coli*) revealed a higher frequency of CK elevation (35%) in comparison with anti-PM/Scl-100-negative patients (11.5%; *P *= 0.009) (Table 3). There was only a tendency of an association between the presence of anti-PM/Scl-100 and the prevalence of lung fibrosis (*P *= 0.086). On the other hand, anti-PM/Scl-100-positive patients suffered less frequently from gastrointestinal involvements such as diarrhoea, regular emesis, or constipation. For instance, only 55% of the anti-PM/Scl-100-positive patients reported episodes of diarrhoea, in contrast to 78.5% of the negative patients (*P *= 0.026). When anti-PM/Scl-100 antibody-positive and -negative patients were compared, no significant differences in the age at disease onset were found (mean ages of 48.3 and 49.8 years, respectively). Fewer clinical associations were detectable when reactivity to the PM/Scl-100 antigen expressed by baculovirus was analyzed. Here, anti-PM/Scl-100 antibodies were associated only with an increase in CK (*P *= 0.043, data not shown).

**Table 3:** Comparison of PM/Scl-100 antibody-positive versus-negative patients

Disease manifestation	anti-PM/Scl-100-positive (n = 20)	anti-PM/Scl-100-negative (n = 261)	*P *value	Sensitivity, percentage^a^	Specificity, percentage^b^
Digital ulcers^c^	12 (60.0)	100 (38.5)	0.095	10.7	95.2
Lung fibrosis^c^	11 (55.0)	87 (33.5)	0.086	11.2	95.1
CK elevation^c^	7 (35.0)	30 (11.5)	0.009	18.9	94.7
Esophago-gastral involvement^c^	11 (55.0)	204 (78.5)	0.026	5.1	86.2
Small intestinal involvement^c^	3 (15.0)	100 (38.5)	0.052	2.9	90.4
Colon involvement^c^	4 (20.0)	115 (44.2)	0.037	3.4	90.1

Clinical differences between antibody-positive and -negative patients were shown only when *P *values of below 0.1 were detected. ^a,b^Calculations of sensitivity and specificity are analogous to those in Table 2. ^c^Values displayed are numbers of patients (percentages). CK, creatine kinase.

## Discussion

Anti-PM/Scl antibodies are supposed to be a marker for overlap syndromes; however, the diagnostic impact of their major subspecificities, anti-PM/Scl-75 and anti-PM/Scl-100, as well as their prevalence in different SSc subsets are still not known. In the present study, a large well-characterized cohort was analyzed for the presence of the different anti-PM/Scl antibodies.

As shown here, anti-PM/Scl antibodies, in particular anti-PM/Scl-75, are more frequent than had previously been described in SSc cohorts. Reactivity to either PM/Scl-100 or PM/Scl-75 was found to depend on the underlying disease subset. Anti-PM/Scl-75 antibodies are found mostly in patients with dSSc and overlap syndromes, whereas anti-PM/Scl-100 antibodies are detected mainly in patients with overlap syndromes. Anti-PM/Scl antibodies were highly specific for SSc; however, our analyses did not include patients with primary PM, dermatomyositis, or inclusion body myositis, conditions that could influence the specificity of the assays.

When the clinical data of the patients were studied, anti-PM/Scl antibody-positive patients significantly more often showed muscle involvement and lung fibrosis, confirming other studies [2,24]. Furthermore, and not described before, digital ulcers were found to be associated with the presence of anti-PM/Scl antibodies. In contrast, PAH was less frequently detected in anti-PM/Scl-positive patients. Anti-PM/Scl-75 antibody-positive patients were younger at disease onset compared with the anti-PM/Scl-75-negative patients. In the presence of anti-PM/Scl-100 antibodies, fewer gastrointestinal symptoms were found. In view of these findings, detection and distinction of both antibody specificities appear to be important beyond the increase in sensitivity for SSc and overlap syndromes.

Only a few studies have analyzed the sensitivity and specificity of anti-PM/Scl antibodies in large SSc cohorts. One of the first studies from the Pittsburgh group identified 23 (4%) of 617 patients with connective tissue diseases as being positive for anti-PM/Scl; this cohort included 314 patients with SSc, 89 patients with overlap syndromes, and 106 patients with pure dermatomyositis/PM. For the identification of the anti-PM/Scl antibodies, immunoprecipitation and immunodiffusion were used. The description of anti-PM/Scl-positive patients revealed a higher frequency of myositis and a lower incidence of kidney involvement. There were no differences in the frequency of pulmonary diseases [4]. However, the number of anti-PM/Scl-positive patients was too small to evaluate significant differences compared with the antibody-negative group. Other studies suggested a higher incidence of muscle involvement, confirming studies analyzing myositis patients with or without SSc overlap [2,7,24,26-28]. Here, we showed the association between the presence of anti-PM/Scl antibodies in SSc patients with myositis and with muscle atrophy. In addition, anti-PM/Scl antibodies are a marker of lung and skin fibrosis and of active disease, as previously described also for SSc patients with anti-topo I antibodies in the same cohort [5,12]. Indeed, a significant proportion of the anti-PM/Scl-positive patients also exhibited reactivity to the topo I autoantigen (35.1%). This subgroup of double-positive patients exhibited higher frequencies of restrictive lung disease (91% versus 46%; *P *= 0.02) and of contractures (92% versus 59%; *P *= 0.049) when compared with the anti-PM/Scl single-positive patients. In this double-positive group, a lower percentage of patients showed CK elevation (23% versus 36%; *P *= 0.02) compared with single-positive patients. In contrast to anti-topo I antibodies, and probably due to the low number of anti-PM/Scl-positive patients, there was no increased mortality related to the signal strengths of the anti-PM/Scl antibodies. However, 4 out of 29 anti-PM/Scl-75-positive patients (13.8%) died within 3 years after antibody detection (3 of them were also positive for anti-topo I antibodies and 2 also exhibited anti-PM/Scl-100 reactivity) compared with 6.1% in our whole SSc cohort. One further patient received autologous stem cell transplantation. This mortality, the co-incidence with anti-topo I antibodies, the disease characteristics with a high frequency of lung fibrosis, and the increased disease activity score especially in anti-PM/Scl-75-positive patients do not support former studies claming a milder disease with a favourable prognosis and response to immunosuppression [5,6].

Here, we could demonstrate for the first time that the reactivity to PM/Scl depends on the underlying disease and furthermore on the clinical symptoms. Interestingly, the majority of patients showed reactivity to only one of the two major autoantigens. Only 37.1% of anti-PM/Scl-positive patients were double-positive for both subsets of antibodies. Nevertheless, the higher prevalence of anti-PM/Scl-75 antibodies and the higher rate of clinical associations indicate that PM/Scl-75 is the main autoantigen in SSc patients. Therefore, when tests are used based on PM/Scl-100 as an antigen source, as most ELISA and LIA techniques are [3], reactivity to the anti-PM/Scl-75 antigen can be missed, especially in dSSc patients. According to our results, both specificities should be determined.

Results concerning the main antigenic targets of anti-PM/Scl antibodies are controversial. Studies analyzing sera from large myositis cohorts ascribed the highest reactivity to the PM/Scl-100 autoantigen [26]. However, this finding might be representative for myositis patients. In an analysis of sera from different disciplines including SSc patients, the PM/Scl-75, especially the major isoform PM/Scl-75c, was considered the main epitope of anti-PM/Scl antibodies [9,10]. Just recently, the PM/Scl-100 epitope-based ELISA (PM1-alpha) was compared with recombinant PM/Scl-100 and PM/Scl-75c [29]. Thus, further studies are mandatory to address diagnostic accuracy of the individual PM/Scl antigens.

Furthermore, reactivity to the PM/Scl antigens seems to be influenced by the system applied for antigen expression. For the PM/Scl-100 antigen, the baculovirus expression system did not provide additional benefit when compared with the *E. coli *expression system. Therefore, post-translational modifications made by eucaryotic cells do not appear to play a role in anti-PM/Scl-100 antibody binding. Consequently, the PM/Scl-100 antigen can be produced using the *E. coli *expression system as an easy and cost-effective method [30,31].

In summary, this is the first report about the prevalence of different anti-PM/Scl antibodies in SSc patients classified and assessed by the commonly used standards of the DNSS and EUSTAR network. Antibodies against PM/Scl-75 and PM/Scl-100 can be considered independent markers for different SSc subsets and show partial differences with respect to associated clinical manifestations, substantiating the diagnostic relevance of their parallel determination.

## Conclusion

The prevalence of anti-PM/Scl-75 and anti-PM/Scl-100 antibodies depends on the underlying SSc subsets and the patients' clinical manifestations. Their presence is not associated with a favourable outcome, especially in the presence of anti-PM/Scl-75 antibodies. Patients with dSSc show reactivity directed mainly to the PM/Scl-75 autoantigen, whereas overlap syndromes can reveal reactivity to PM/Scl-75 and PM/Scl-100. A major proportion of SSc patients might remain undetected when applied tests are limited to the detection of anti-PM/Scl-100 antibodies.

## Abbreviations

anti-topo I: anti-topoisomerase I; CENP-B: centromere protein-B; CK: creatine kinase; DLCO-SB: predicted diffusion capacity of a single breath; DNSS: German Network (Deutsches Netzwerk) of Systemic Scleroderma; dSSc: diffuse systemic sclerosis; ELISA: enzyme-linked immunosorbent assay; EUSTAR: European Scleroderma Trials and Research; FVC: forced vital capacity; LIA: line immunoblot assay; lSSc: limited systemic sclerosis; mRSS: modified Rodnan skin score; PAH: pulmonary arterial hypertension; PM: polymyositis; RA: rheumatoid arthritis; RP: Raynaud phenomenon; SD: standard deviation; SLE: systemic lupus erythematosus; SS: Sjögren syndrome; SSc: systemic sclerosis; SScSS: systemic sclerosis sine scleroderma; U: units; UCTD: undifferentiated connective tissue disease.

## Competing interests

GR has received fees from EUROIMMUN AG for lectures on these data at the 'Eurodoctor' meeting in Brussels, Belgium. KH was invited by EUROIMMUN AG to participate in a national meeting to show the results of the study. After finishing the study, she received a grant from EUROIMMUN AG for additional scientific work. The other authors declare that they have no competing interests.

## Authors' contributions

KH and AK helped to provide preclinical analyses, statistics, and graphics and to write the manuscript. CD, AJ, LK, and WM helped to develop the LIA and to perform the tests. M Backhaus provided access to the patients in her outpatient department. CSB helped to provide clinical data. M Becker corrected and helped to write the manuscript. DH supervised statistical analyses. KE participated in discussions of the data with GR, made intellectual contributions, helped to prepare the manuscript, and provided sera for the analyses. GRB and FH participated in discussions of the data with GR, made intellectual contributions, and helped to prepare the manuscript. WS organized all of the cooperation with EUROIMMUN AG and made intellectual contributions. GR, as the author responsible for this report, initiated this study and controlled the work. She collected and assessed the patients, helped to provide clinical data, and wrote and reviewed the manuscript. All authors read and approved the final manuscript.

## References

[B1] Reichlin M, Maddison PJ, Targoff I, Bunch T, Arnett F, Sharp G, Treadwell E, Tan EM (1984). Antibodies to a nuclear/nucleolar antigen in patients with polymyositis overlap syndromes. J Clin Immunol.

[B2] Steen VD (2005). Autoantibodies in systemic sclerosis. Semin Arthritis Rheum.

[B3] Mahler M, Raijmakers R (2007). Novel aspects of autoantibodies to the PM/Scl complex: clinical, genetic and diagnostic insights. Autoimmun Rev.

[B4] Oddis CV, Okano Y, Rudert WA, Trucco M, Duquesnoy RJ, Medsger TA (1992). Serum autoantibody to the nucleolar antigen PM-Scl. Clinical and immunogenetic associations. Arthritis Rheum.

[B5] Marguerie C, Bunn CC, Copier J, Bernstein RM, Gilroy JM, Black CM, So AK, Walport MJ (1992). The clinical and immunogenetic features of patients with autoantibodies to the nucleolar antigen PM-Scl. Medicine (Baltimore).

[B6] Vandergheynst F, Ocmant A, Sordet C, Humbel RL, Goetz J, Roufosse F, Cogan E, Sibilia J (2006). Anti-pm/scl antibodies in connective tissue disease: clinical and biological assessment of 14 patients. Clin Exp Rheumatol.

[B7] Alderuccio F, Chan EK, Tan EM (1991). Molecular characterization of an autoantigen of PM-Scl in the polymyositis/scleroderma overlap syndrome: a unique and complete human cDNA encoding an apparent 75-kD acidic protein of the nucleolar complex. J Exp Med.

[B8] Blüthner M, Bautz FA (1992). Cloning and characterization of the cDNA coding for a polymyositis-scleroderma overlap syndrome-related nucleolar 100-kD protein. J Exp Med.

[B9] Raijmakers R, Renz M, Wiemann C, Egberts WV, Seelig HP, van Venrooij WJ, Pruijn GJ (2004). PM-Scl-75 is the main autoantigen in patients with the polymyositis/scleroderma overlap syndrome. Arthritis Rheum.

[B10] Raijmakers R, Egberts WV, van Venrooij WJ, Pruijn GJ (2003). The association of the human PM/Scl-75 autoantigen with the exosome is dependent on a newly identified N terminus. J Biol Chem.

[B11] Mahler M, Raijmakers R, Dähnrich C, Blüthner M, Fritzler MJ (2005). Clinical evaluation of autoantibodies to a novel PM/Scl peptide antigen. Arthritis Res Ther.

[B12] Hanke K, Dahnrich C, Bruckner C, Huscher D, Becker M, Janssen A, Meyer W, Egerer K, Hiepe F, Burmester G R, Schlumberger W, Riemekasten G (2009). Diagnostic value of anti-topoisomerase I antibodies in a large monocentric cohort. Arthritis Res Ther.

[B13] Walker UA, Tyndall A, Czirják L, Denton C, Farge-Bancel D, Kowal-Bielecka O, Müller-Ladner U, Bocelli-Tyndall C, Matucci-Cerinic M (2007). Clinical risk assessment of organ manifestations in systemic sclerosis: a report from the EULAR Scleroderma Trials And Research group database. Ann Rheum Dis.

[B14] Hunzelmann N, Genth E, Krieg T, Lehmacher W, Melchers I, Meurer M, Moinzadeh P, Müller-Ladner U, Pfeiffer C, Riemekasten G, Schulze-Lohoff E, Sunderkoetter C, Weber M, Worm M, Klaus P, Rubbert A, Steinbrink K, Grundt B, Hein R, Scharffetter-Kochanek K, Hinrichs R, Walker K, Szeimies RM, Karrer S, Müller A, Seitz C, Schmidt E, Lehmann P, Foeldvári I, Reichenberger F (2008). Registry of the German Network for Systemic Scleroderma. The registry of the German Network for Systemic Scleroderma: frequency of disease subsets and patterns of organ involvement. Rheumatology (Oxford).

[B15] Czirják L, Nagy Z, Aringer M, Riemekasten G, Matucci-Cerinic M, Furst DE, EUSTAR (2007). The EUSTAR model for teaching and implementing the modified Rodnan skin score in systemic sclerosis. Ann Rheum Dis.

[B16] LeRoy EC, Black C, Fleischmajer R, Jablonska S, Krieg T, Medsger TA, Rowell N, Wollheim F (1988). Scleroderma (systemic sclerosis): classification, subsets and pathogenesis. J Rheumatol.

[B17] Bennett RM (1990). Scleroderma overlap syndrome. Rheum Dis Clin North Am.

[B18] Rodnan GP, Fennel RH (1962). Progressive systemic sclerosis sine scleroderma. JAMA.

[B19] Mosca M, Neri R, Bombardieri S (1999). Undifferentiated connective tissue diseases (UCTD): a review of the literature and a proposal for preliminary classification criteria. Clin Exp Rheumatol.

[B20] Vitali C, Bombardieri S, Moutsopoulos HM, Balestrieri G, Bencivelli W, Bernstein RM, Bjerrum KB, Braga S, Coll J, de Vita S, Drosos AA, Ehrenfeld M, Hatron PY, Hay EM, Isenberg DA, Janin A, Kalden JR, Kater L, Konttinen YT, Maddison PJ, Maini RN, Manthorpe R, Meyer O, Ostuni P, Pennec Y, Prause JU, Richards A, Sauvezie B, Schiødt M, Sciuto M (1993). Preliminary criteria for the classification of Sjögren's syndrome. Results of a prospective concerted action supported by the European Community. Arthritis Rheum.

[B21] Tan EM, Cohen AS, Fries JF, Masi AT, McShane DJ, Rothfield NF, Schaller JG, Talal N, Winchester RJ (1982). The 1982 revised criteria for the classification of systemic lupus erythematosus. Arthritis Rheum.

[B22] Arnett FC, Edworthy SM, Bloch DA, McShane DJ, Fries JF, Cooper NS, Healey LA, Kaplan SR, Liang MH, Luthra HS, Medsger TA, Mitchell DM, Neustadt DH, Pinals RS, Schaller JG, Sharp JT, Wilder RL, Hunder GG (1988). The American Rheumatism Association 1987 revised criteria for the classification of rheumatoid arthritis. Arthritis Rheum.

[B23] Furst DE, Clements PJ, Steen VD, Medsger TA, Masi AT, D'Angelo WA, Lachenbruch PA, Grau RG, Seibold JR (1998). The modified Rodnan skin score is an accurate reflection of skin biopsy thickness in systemic sclerosis. J Rheumatol.

[B24] Valentini G, Bencivelli W, Bombardieri S, D'Angelo S, Della Rossa A, Silman AJ, Black CM, Czirjak L, Nielsen H, Vlachoyiannopoulos PG (2003). European Scleroderma Study Group to define disease activity criteria for systemic sclerosis. III. Assessment of the construct validity of the preliminary activity criteria. Ann Rheum Dis.

[B25] Blüthner M, Mahler M, Müller DB, Dünzl H, Bautz FA (2000). Identification of an alpha-helical epitope region on the PM/Scl-100 autoantigen with structural homology to a region on the heterochromatin p25beta autoantigen using immobilized overlapping synthetic peptides. J Mol Med.

[B26] Selva-O'Callaghan A, Labrador-Horrillo M, Solans-Laque R, Simeon-Aznar CP, Martínez-Gómez X, Vilardell-Tarrés M (2006). Myositis-specific and myositis-associated antibodies in a series of eighty-eight Mediterranean patients with idiopathic inflammatory myopathy. Arthritis Rheum.

[B27] Brouwer R, Vree Egberts WT, Hengstman GJ, Raijmakers R, van Engelen BG, Seelig HP, Renz M, Mierau R, Genth E, Pruijn GJ, van Venrooij WJ (2002). Autoantibodies directed to novel components of the PM/Scl complex, the human exosome. Arthritis Res.

[B28] Brouwer R, Hengstman GJ, Vree Egberts W, Ehrfeld H, Bozic B, Ghirardello A, Grøndal G, Hietarinta M, Isenberg D, Kalden JR, Lundberg I, Moutsopoulos H, Roux-Lombard P, Vencovsky J, Wikman A, Seelig HP, van Engelen BG, van Venrooij WJ (2001). Autoantibody profiles in the sera of European patients with myositis. Ann Rheum Dis.

[B29] Mahler M, Mierau R, Humbel RL, Fritzler MJ Anti-PM1-alpha antibodies: analytical evaluation in two centers.

[B30] Schmidt M, Hoffman DR (2002). Expression systems for production of recombinant allergens. Int Arch Allergy Immunol.

[B31] Hu YC (2005). Baculovirus as a highly efficient expression vector in insect and mammalian cells. Acta Pharmacol Sin.

